# Sparse orthogonal population representation of spatial context in the retrosplenial cortex

**DOI:** 10.1038/s41467-017-00180-9

**Published:** 2017-08-15

**Authors:** Dun Mao, Steffen Kandler, Bruce L. McNaughton, Vincent Bonin

**Affiliations:** 1 Neuro-Electronics Research Flanders, Kapeldreef 75, Leuven, B-3001 Belgium; 2 Canadian Centre for Behavioural Neuroscience, Department of Neuroscience, University of Lethbridge, 4401 University Drive, Lethbridge, Canada AB T1K 3M4; 3 Imec, Kapeldreef 75, Leuven, B-3001 Belgium; 4 VIB, Leuven, 3001 Belgium; 5 Department of Biology, KU Leuven, Naamsestraat 59, Leuven, 3000 Belgium

## Abstract

Sparse orthogonal coding is a key feature of hippocampal neural activity, which is believed to increase episodic memory capacity and to assist in navigation. Some retrosplenial cortex (RSC) neurons convey distributed spatial and navigational signals, but place-field representations such as observed in the hippocampus have not been reported. Combining cellular Ca^2+^ imaging in RSC of mice with a head-fixed locomotion assay, we identified a population of RSC neurons, located predominantly in superficial layers, whose ensemble activity closely resembles that of hippocampal CA1 place cells during the same task. Like CA1 place cells, these RSC neurons fire in sequences during movement, and show narrowly tuned firing fields that form a sparse, orthogonal code correlated with location. RSC ‘place’ cell activity is robust to environmental manipulations, showing partial remapping similar to that observed in CA1. This population code for spatial context may assist the RSC in its role in memory and/or navigation.

## Abstract (web summary)

Neurons in the retrosplenial cortex (RSC) encode spatial and navigational signals. Here the authors use calcium imaging to show that, similar to the hippocampus, RSC neurons also encode place cell-like activity in a sparse orthogonal representation, partially anchored to the allocentric cues on the linear track.

## Introduction

As early as 1969, Marr pointed out that making neural codes sparse (minimizing the proportion of active units) and orthogonal (making codes as statistically uncorrelated as possible) maximizes the number of different patterns that can be stored in associative memory networks such as the cerebellar cortex, hippocampus, and neocortex. A prime example of such a code is neural activity in the rodent hippocampus during spatially extended behaviour. At the neurophysiological level, hippocampal neurons show discrete patches of high-rate discharges localized to specific places^1^, which are referred to as place fields. The activity of hippocampal place cells is characterized by overall low probability of activation, and precise tuning and a nearly uniform allocation of place fields over the environment. The integration of self-motion cues (path integration) has been shown to be sufficient for the generation of hippocampal place cells; however, the path integration mechanism appears to require frequent correction from external cues^2^. Computationally, place-cell activity can be viewed as basis vectors upon which the animal’s spatial location and other contextual events are encoded^3^. Place cell activity is sparse and approximately orthogonal (statistically uncorrelated)^4^, features that are thought to be important for spatial learning and memory, route planning, and navigation^1, 5–8^.

Is the hippocampus unique in its sparse contextual coding scheme? Considering the sparse firing of neurons in the superficial neocortex^9^, it is possible that similar orthogonal codes of location are expressed in immediately interconnected cortical regions^10^. Notably, the retrosplenial cortex (RSC) is densely connected with the hippocampal system^11–13^, including direct input from dorsal hippocampal CA1. Moreover, the RSC has been directly linked to spatial behaviours. For example, inactivating the RSC impairs spatial behaviours^14^ and path integration in darkness^15^. Likewise, lesions of the RSC produce spatial and contextual memory deficits that are similar to those observed after hippocampal lesions^14, 16–21^. Studies of RSC neurons during spatially extended behaviour have shown navigation-related signals, including head-direction selective, speed selective and goal-related signals^22–24^. Recent studies have also shown evidence of route encoding^25–27^ but reported only broad spatial and behavioural selectivity^23, 25^. To date, few studies have achieved simultaneous recordings from a sufficiently large number of neurons to characterize the RSC population code.

The purpose of the present investigation was therefore to characterize population activity in the RSC during spatially extended behaviour. We performed cellular Ca^2+^ imaging across subregions of the RSC in mice that used natural locomotion to move a treadmill linear track. We discovered a substantial group of RSC neurons, most numerous in the superficial subregions, that show spatially-localized activity that closely resembles the activity of CA1 place cells measured in the same behavioural assay. Like CA1 place cells, these RSC neurons fire in sequences during movement, and show narrowly tuned firing fields that form a sparse, orthogonal representation of the environment. RSC ‘place’ cell activity is robust to environmental manipulations, showing partial random remapping of its activity. This RSC place code may be useful for spatially guided behaviour such as goal-directed navigation, and for memory encoding in general. Whether this hippocampus-like activity is independent of the hippocampus, inherited from it, or helps shape it will require further investigation.

## Results

### Hippocampal place cell activity during treadmill locomotion

**Fig. 1:**
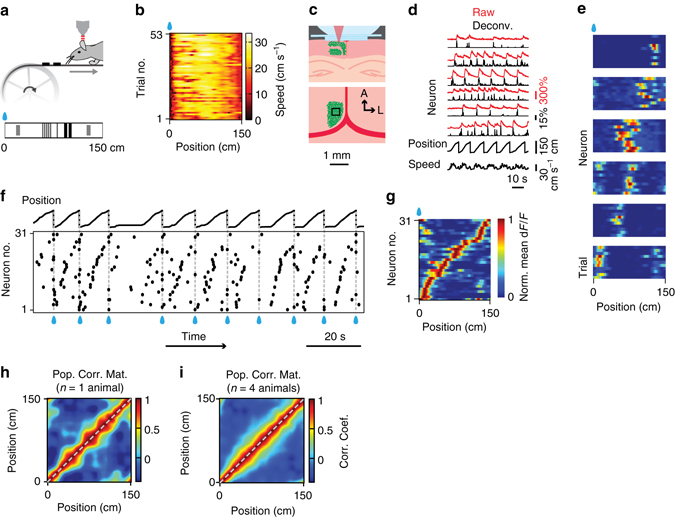
Sparse orthogonal population representation of spatial location in the retrosplenial cortex. **a** Head-fixed locomotion assay. Mice moved a 150-cm linear treadmill (*top*) with tactile cues on its surface (*bottom*). A drop of sucrose water (*blue*) was delivered at a fixed location for every completed lap. **b** Lap running behaviour. Movement speed as a function of location for 53 consecutive laps from one experimental session. The animal moved robustly and slowed down or paused most frequently near the reward (as shown by *dark colours* on the *left*). **c** Cellular imaging of neural activity in the retrosplenial cortex (RSC) during head-fixed treadmill running. (*top*) Illustration of superficial and deep RSC neurons labelled with calcium indicator GCaMP6m (green dots). Calcium imaging was performed with a two-photon microscope through a glass window. (*bottom*) Tangential view of the labelled superficial RSC neurons with an example imaging field of view (*black square*). Red lines indicate superior sagittal sinus and transverse sinuses. Scale bar, 1 mm. A: anterior; L: lateral. **d** Calcium fluorescence signals (*top*, *red*) and inferred neural activity (*top*, *black*) of six example superficial agranular RSC neurons showing place cell activity; speed and treadmill position are at the bottom. Neural activity was inferred using a fast non-negative deconvolution algorithm 64. **e** Normalized activity of the six RSC place cells in **d** as a function of location for multiple laps. The *y* axis in each colour map corresponds to trial number. Note how neurons were activated as the animal crossed specific locations. Activity was normalized to the time spent at individual locations. **f** Raster plot showing activation time points for 31 simultaneously imaged RSC place cells, for the same session as in **e**, together with position (*top*). Activation time points defined as time points of peak response in each lap for each neuron. Cells ordered by the location that evoked largest responses. Note the repeated sequences of activation during movement and lack of activation when the animal was not moving. **g** Average normalized activity as a function of location for the 31 RSC place cells shown in **f**. **h** Correlation matrix (Pearson correlation coefficient) of population vectors as a function of position for data shown in **g**. **i** Correlation matrix (Pearson correlation coefficient) of population vectors as a function of position for data from four mice. (Data from WT mice with AAV1-hSyn-GCaMP6m injections.)

To study spatial coding in the RSC, we combined a head-fixed locomotion assay^28^ with chronic cellular imaging (Fig. 1a; see also Methods). C57Bl/6j mice (*n* = 14 wild-type (WT); *n* = 4 Thy1 GP4.3 transgenic mice^29^) were trained to run on a treadmill with tactile stimulus patches on its surface and a fixed reward site (Supplementary Fig. 1a). The animals alternated between high-speed movement and pauses, which were mostly but not exclusively near the reward location (Fig. 1b). Movement speed was approximately constant in between reward events (18.5 ± 5.5 cm s^−1^, mean ± s.d.; *n* = 18 mice, 40 runs). Experimental sessions lasted 11.3 ± 2.8 min (mean ± s.d.), yielding 33 ± 17 (mean ± s.d.) complete laps (trials) per session.

We expected that treadmill running would entrain neural activity in the hippocampus^28^ as observed in freely moving animals. To confirm this, we measured cellular activity in dorsal CA1 using chronic cellular imaging^30, 31^ (*n* = 1758 neurons, *n* = 2 WT mice; *n* = 1 Thy1 transgenic mice) and multisite silicon probe recordings (*n* = 337 neurons, *n* = 8 mice) (Supplementary Fig. 1b, e). Consistent with studies in freely moving animals^4^, CA1 neuronal activity was sparse (calcium transient rate = 0.8, 2.6, 11.3 min^−1^; 10, 50 and 90% percentiles) and the distribution of firing rates of isolated single units was skewed (10, 50 and 90% percentiles of firing rate = 0.6, 1.8 and 4.7 spikes s^−1^).

To assess spatial modulation of neuronal activity, calcium time courses were deconvolved^32^ (Fig. 1d) and expressed as a function of the animal’s location on the treadmill, in 1.5 cm intervals, and normalized by the time spent at each location. We used the resulting position-related activity profiles as measures of spatial tuning. Similar to cellular imaging measurements made in real^4^ and virtual^28, 30^ environments, 47% (159/337) of electrically recorded and 26% (452/1758) of optically recorded hippocampal CA1 neurons met established criteria for place cell activity (see Methods) (Supplementary Fig. 1c, f)^30, 33^. The active neurons showed sequential firing during movement (Supplementary Fig. 1g) and discrete, minimally-overlapping place fields that continuously, but sparsely, covered the treadmill belt (Supplementary Fig. 1d, h)^1, 28^. In the electrical recordings, place-cell activity was phase locked to low frequency local-field potential oscillations (5–12 Hz; Supplementary Fig. 1c), as observed in freely moving animals^34, 35^. Thus, running on the treadmill entrains hippocampal CA1 activity in a way that resembles that observed during free running in linear environments.

### Spatial activity in the retrosplenial cortex

Next, we used the head-fixed treadmill assay to investigate neural correlates of spatial location in the RSC. We first examined the calcium time courses of 2256 (*n* = 4 WT mice, 14 sessions) neurons in the superficial agranular region of RSC. The neurons were labelled via AAV1-hSyn-GCaMP6m viral vector injections and imaged with a two-photon microscope through a cranial glass window implanted over the midline^31^ (Fig. 1c). To study spatial tuning, we expressed neuronal activity as a function of the animal’s location on the treadmill and averaged the result across laps, using the same procedure used for the CA1 recordings (see Methods).

**Fig. 2:**
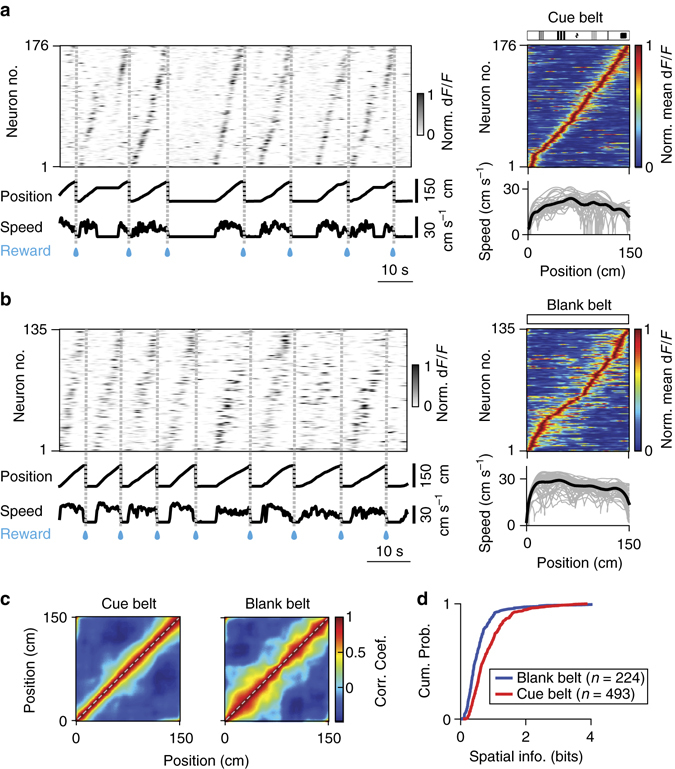
Tactile stimuli enhance stability of RSC place cell activity. **a** (*left*) Normalized calcium activity of 176 simultaneously imaged RSC place cells on a belt with tactile cues. Cells were ordered by the positions of their peak average activity. Position and speed traces are shown below. *Dashed lines* and *blue drops* represent reward delivery. (*right*) trial-averaged position activity for the 176 RSC place cells shown on the *left*. Belt diagram (*top*) and speed traces as a function of position (*bottom*) are shown. *Grey lines*, speed traces for individual trials; *black line*, average speed trace. **b** Same, for 135 neurons imaged during movement on a belt devoid of salient tactile cues. Note the increased positional jitter of RSC place-cell activity in absence of salient tactile stimuli. **c** Correlation matrices (Pearson correlation coefficient) of population vectors as a function of position for RSC place cells on the cue belt (*left*) and on the blank belt (*right*). **d** Cumulative distributions of spatial information for all RSC place cells on the cue belt (*red*) and on the blank belt (*blue*). (Data from Thy1 GP4.3 transgenic mice.)

About 13% (297/2256) of RSC neurons labelled with AAV1 viral vector injections had discrete spatially-localized firing fields that resembled the place fields of CA1 neurons. Accordingly, we refer to these cells as RSC place cells. Similar to CA1 place cells (Supplementary Fig. 1d, g, h), the RSC place cells showed spatially localized activity with distinct neurons firing as the animal crossed distinct treadmill locations (Fig. 1e). Within simultaneously imaged neuronal populations, the firing of RSC place neurons sparsely but continuously covered the full length of the treadmill (Fig. 1e–g). The RSC neurons fired in continuous, reproducible sequences during movement but not during stillness (Figs 1f and 2a). Similar results were obtained in Thy1 GP4.3 transgenic mice (*n* = 3)^29^ (Fig. 2), which specifically express GCaMP6 in a subset of cortical excitatory neurons. In these mice, the fraction of place cells was larger (56%, 1416/2544, *n* = 3 mice, eight sessions), possibly due to a bias in favour of a specific group of excitatory neurons. Importantly, there were many active cells in the RSC whose activity did not show place-cell characteristics. The characteristics of these ‘non-place’ cells will be reported elsewhere.

### Similarity of the RSC and CA1 place codes

**Fig. 3:**
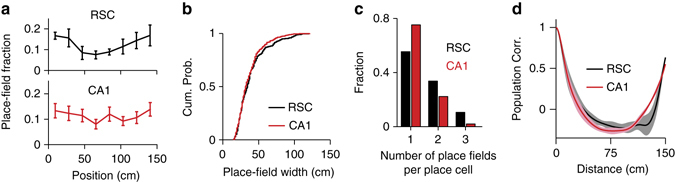
Similar spatial response properties of RSC and CA1 place cells. **a** Place-field fraction as a function of place field location on the track. (*top*) RSC place cells (*n* = 297); bottom: CA1 place cells (*n* = 611, electrophysiology and imaging). Error bars: s.e.m. **b** Cumulative probability distributions of place-field widths for RSC place cells (*black*, *n* = 297) and CA1 place cells (*red*, *n* = 452, imaging). **c** Distribution of place field count per cell for RSC (*black bars*, *n* = 297) and CA1 (*red bars*, *n* = 611, electrophysiology and imaging) place cells. **d** Population vector correlation (Pearson correlation coefficient) as a function of distance for RSC (*black*) and CA1 (*red*) place cells. Shaded areas represent s.d. Note that the periodicity occurred because of the periodicity of the track. (Data from WT mice with AAV1-hSyn-GCaMP6m injections.)

The spatial tuning properties of RSC and CA1 place cells were strikingly similar. RSC and CA1 neurons did not differ in the number (Fig. 3c; *P* = 0.21, χ^2^-test) and width of place fields (RSC: 39.6 ± 1.2 cm; CA1: 37.0 ± 0.8 cm; both mean ± s.e.m.; *P* = 0.57, two-sample KS test) (Fig. 3b). In both areas, place fields were distributed over the length of the treadmill; and there was a tendency of higher density of place fields around the reward location (Fig. 3a). This is sometimes observed in CA1 of freely moving animals^34^.

To quantify spatial tuning at the level of cell populations, we computed the correlation matrix of the neurons’ position-related activity vectors sorted by their peak location, which quantifies response similarity between cells tuned to distinct locations. Consistent with a sparse orthogonal code, RSC population vectors showed high correlations between nearby locations (i.e., near the main diagonal) and weak correlations between more distant locations (Fig. 1h, i). Averaging the data across positions, the neuronal population vectors showed a steep drop in correlation with distance (Fig. 3d). The correlation structure of RSC activity closely resembled that of CA1 neurons during the same behaviour (Fig. 3d and Supplementary Fig. 1i) and during free movement on real linear tracks^35^.

Thus, RSC neurons during linear treadmill running show highly tuned spatial activity that resemble the sparse, orthogonal code of location generally observed in the hippocampus and is characteristic of CA1 neurons’ activity measured during the same behaviour.

### Place-cell activity across subregions of the RSC

**Fig. 4:**
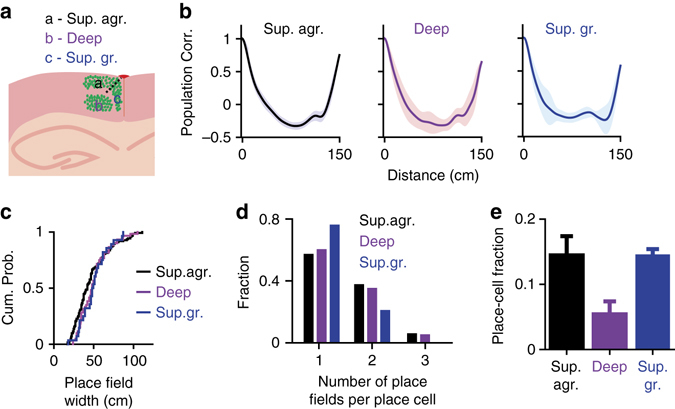
RSC place cells are more prevalent in superficial layers. **a** Diagram of three RSC sub-regions. Sup. agr.: superficial agranular; Deep: deep agranular and granular; Sup. gr.: superficial granular. *Green dots* represent GCaMP6m labelled neurons. Imaging at different depths revealed neurons in different sub-regions. **b** Mean population vector correlation as a function of distance for the three sub-regions. *Shaded areas* represent s.d. **c** Cumulative probability distributions of place-field widths for superficial agranular (*black*, *n* = 123), deep (*purple*, *n* = 60), and superficial granular (*blue*, *n* = 30) RSC place cells. **d** Distribution of place field count per cell for superficial agranular (*black bars*), deep (*purple bars*) and superficial granular (*blue bars*) RSC place cells. **e** Place cell fraction in superficial agranular (*black bar*), deep (*purple bar*), and superficial granular (*blue bar*) RSC. Error bars: s.e.m. (Data from WT mice with AAV1-hSyn-GCaMP6m injections.)

Distinct subregions of the RSC receive distinct inputs and make synaptic connections to distinct targets^11, 36, 37^, which could indicate functionally specialized domains. To compare spatial activity across subregions of the RSC, we imaged in each animal (*n* = 3 WT mice) neurons at two distinct depths (150 and 400 µm) and used the lack of GCaMP6 expression in layer 4 to determine the boundaries of RSC subregions in the in vivo images (Fig. 4a and Supplementary Fig. 2a). While hippocampus-like place cells were observed across all subregions of the RSC (Supplementary Fig. 2b), they were substantially more prevalent in superficial regions (agranular and granular). Place cells were found in the superficial agranular (*n* = 123/861), superficial granular (*n* = 31/198) and deep layers (*n* = 60/1237) of the RSC. A similar steep drop in population activity correlation with distance was observed in the three RSC subregions (Fig. 4b). Place cells across RSC subregions had similar spatial response properties (Figs. 4c and d; *P* = 0.58 for place-field width comparison, One-way analysis of variance; *P* = 0.27 for place field number comparison, χ^2^-test); however, there were about 2.5 times more place cells in the superficial agranular and superficial granular layers than the deep layers (superficial agranular: 15 ± 3%; superficial granular: 15 ± 1%; deep layers: 6 ± 2 %; mean ± s.e.m.; superficial vs. deep, both *P* < 0.05, paired *t*-test) (Fig. 4e). These results are consistent with the anatomical connections between dorsal hippocampus CA1 and superficial but not deep RSC^12^.

### Tactile stimuli enhance stability of RSC place-cell activity

In CA1, place fields in absence of ‘allocentric’ cues from external stimuli reflect path integration between reward events^38, 39^. Place-cell activity in the RSC could depend on the integration of self-motion cues (that is, from belt and limb movement), but, if so, would require anchoring to external stimuli, of which only the reward delivery and the tactile cues on the belt were available. To assess the specific contribution of tactile inputs, we compared activity on treadmill belts with and without tactile stimuli. Mice were well-trained in both conditions. We compared the impact of tactile stimuli on CA1 (*n* = 1 Thy1 transgenic mouse) and RSC (*n* = 3 Thy1 transgenic mice) activity.

Place-cell activity in the hippocampus was maintained in the reference frame of the reward stimulus in absence of tactile stimuli (Supplementary Fig. 3). CA1 neurons showed seamless sequential firing during movement on belts with and without tactile stimuli (Supplementary Fig. 3a, b). The tactile stimuli had little impact on population spatial tuning, showing the same rapid decrease in correlation with distance in presence and absence of tactile stimuli (Supplementary Fig. 3c). Spatial information (SI) encoded in the neurons’ activities was also similar, differing by <10% between the two conditions (Supplementary Fig. 3d; SI: cue belt, 0.93 ± 0.02; blank belt, 0.86 ± 0.03, both mean ± s.e.m.; *P* = 0.03, two-sample *t*-test).

Place-cell activity in absence of tactile cues was also maintained in RSC (Fig. 2). On the treadmill devoid of tactile stimuli, RSC neural population showed sequential firing (Fig. 2b) and similar spatial tuning (Fig. 2c). However, RSC neurons showed more pronounced trial-to-trial position jitter in the absence of tactile stimuli (Fig. 2b). This resulted in reduced tuning of the population (Fig. 2c) and lower SI in the neurons’ activity (Fig. 2d; SI: cue belt, 0.86 ± 0.03, mean ± s.e.m., *n* = 493; blank belt, 0.61 ± 0.04, mean ± s.e.m., *n* = 224; *P* = 2.4e-07, two-sample *t*-test).

Thus, while tactile stimuli were not required for place cell activity in RSC, tactile stimuli enhanced the reliability of the RSC spatial representation somewhat more than in the hippocampus. Although the place-cell activities observed in CA1 and RSC during treadmill running are similar, the more pronounced impact of manipulating tactile stimuli suggests that RSC responses may depend more on external sensory inputs; however, since there were few mice, we cannot rule out a contribution from between-animal differences.

### RSC population encodes ‘allocentric’ treadmill position

Do RSC neurons encode location on the treadmill independent of task context? In the hippocampus, place fields rearrange seemingly unpredictably upon changes of environment and/or task context^40–42^, a process referred to as place-field remapping. Within a single environment, in the hippocampus, neurons rarely remap their place fields to different locations in response to external cue manipulations; most maintain their place fields at the same location but may adjust their in-field firing rates. In some cases this ‘rate-remapping’ is strong enough that, without adjustment of plotting scale, a few fields may seem to appear or disappear^41^. These firing rate changes allow the hippocampus to encode uniquely the conjunction of location and the behaviours and experiences that occur at a given location.

To probe remapping in the RSC, we examined, in separate experiments, the impact of changes of room illumination and changes of the reward site location on activity in RSC and CA1. The RSC receives strong inputs from the visual cortex^43^ and even shows visual responses^44^. The reward site location impacts running trajectories (Figs 1b and 2a, b) on the treadmill and serves as a salient and reliable event that anchors path integration in the hippocampus. These two factors could influence RSC activity. We compared place-cell activity under photopic illumination and in complete darkness, as well as activity with reward delivered at either of two locations. In all experiments, the belt bore tactile stimuli.

**Fig. 5:**
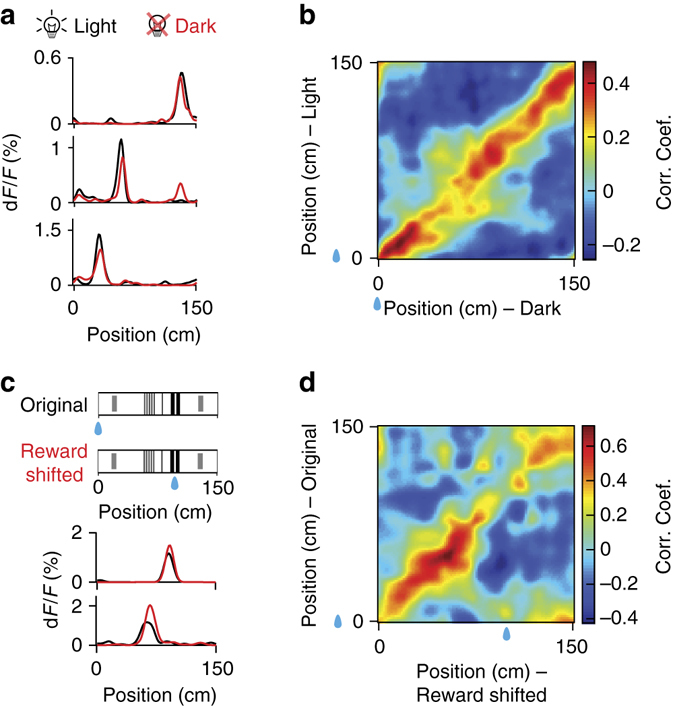
Correlated RSC population representations of position upon environmental illumination change and reward relocation. **a** Spatial tuning curves of three example RSC place cells showing preserved place fields under light (*black*) and dark (*red*) conditions. **b** Population vector correlation (Pearson correlation coefficient) matrix for all RSC place cells under the light and dark conditions. *Blue drops* indicate reward locations. **c** (*top*) Diagram of the reward shift experiment. (*bottom*) Spatial tuning curves of two example RSC place cells showing preserved place fields when the reward site was shifted. *Black lines*, spatial tuning curves before reward shift; *red lines*, spatial tuning curves after reward shift. *Blue drops* indicate reward locations. **d** Population vector correlation (Pearson correlation coefficient) matrix for all RSC place cells under original and reward shifted conditions. *Blue drops* indicate reward locations. (Data from Thy1 GP4.3 transgenic mice **a**, **b** and WT mice with AAV1-hSyn-GCaMP6m injections **c**, **d**.)

RSC place-cell activity was robust to changes in illumination (Fig. 5a and Supplementary Fig. 4a). To compare place-cell activity we computed the neurons preferred locations as well as the correlation of the position-related population activity profiles. RSC place-cell activity was highly correlated across light and dark conditions (Fig. 5b and Supplementary Fig. 4a). However, a small fraction of the neurons showed rearrangement of their place fields (Supplementary Fig. 4a). Similar results were obtained in CA1 (Supplementary Fig. 4b).

Likewise, RSC place-cell activity was also robust to changes in reward site location (Fig. 5c). RSC place-cell activity may encode location in the reference frame defined by the tactile cues, or it may encode position relative to the reward site^45, 46^, possibly by path integration, as in hippocampus^38, 39^. However, RSC place fields were mostly preserved when the reward was shifted (Fig. 5c and Supplementary Fig. 5a). The population representations of position before and after change in reward location were highly correlated (Fig. 5d). However, a number of place cells moved their place fields to near the location of the reward site (Supplementary Fig. 5a). Similar results were obtained in CA1 (Supplementary Fig. 5b).

We conclude that, in presence of spatially-informative tactile stimuli, most RSC neurons encode location in the spatial frame defined by the tactile stimuli. This activity is similar to CA1 place-cell activity measured during the same behavioural assay. It is not driven by eye inputs and is only weakly affected by contextual cues such as the reward site location. However, a fraction of the cells respond to contextual changes by moving their place fields. This could allow the RSC population to distinguish between distinct contexts.

## Discussion

The results indicate that a subpopulation of neurons in the mouse RSC express a sparse, orthogonal and continuous representation of a linear environment that is highly similar in its properties to population of CA1 place cells. As shown in Figs 1 and 2, the representation is sparse because only a small fraction of the cells is active at any one location. As shown in Figs 3 and 4, the code is orthogonal, because, regardless of what the sensory input is, the correlation between population vectors steeply falls to zero over a short distance (~35 cm). In contrast with the implication of the term ‘place cell’; however, in neither brain areas are the place fields irrevocably linked to allocentric position on the track. When spatial context is altered, some neurons show place fields that follow discrete external cues, some remain unchanged and some remap unpredictably. Nevertheless, it is implausible that such discrete place fields could be generated exclusively by external sensory cues, which vary widely in spatial extent and redundancy, and abundant evidence indicates that, in the hippocampus at least, the initial determinant of place field firing comes from path integration mechanisms probably of medial entorhinal origin^2^, followed by associative binding to external patterns of sensory input. Whether such a sparse, orthogonal population code in RSC is generated by a similar mechanism independently of CA1 or whether it is inherited from CA1 remains to be determined, but the fact that place cells were most prevalent in the CA1 recipient layers of RSC (superficial layers) is consistent with the later possibility^12^. To date, the only other non-hippocampal structure to exhibit hippocampus-like place fields in two-dimensional space is the claustrum, which also receives direct CA1 input^47^; however, similar response patterns have been reported in one-dimensional tracks in the prefrontal cortex, which, like RSC, receives strong hippocampal output, and in dorsal striatum^48, 49^.

A role of landmark-referenced path integration in setting up the place-localized firing patterns in the RSC is consistent with human studies which suggest that the RSC is important for path integration with reference to a home location^50, 51^. Consistent with hippocampal place cells, external sensory cues are not needed for RSC place field expression but do help stabilize the overall code over trials.

In general, the sparse, orthogonal and yet flexible characteristics of hippocampal place cells have been considered as optimal for both maximizing information storage locally^52, 53^ and for providing the cortical output targets with a contextual or ‘index’ code to link attributes of a given experience distributed widely over the cortex^53–57^. It is possible that RSC also contributes a similar, partially redundant function or, indeed that it acts as a relay for this ‘index code’ from the hippocampus. Why do such potential index cells appear in the superficial but not the deep layers? One possibility, suggested by Burke et al.^58^, is that the meaningful content (or ‘attributes’) of a cortical memory module is reflected in the activity of deep layer cells, whereas the associations that link these attributes in an experience specific manner are stored in the superficial layers, which would thus maximize event storage capacity by implementing this sort of sparse and orthogonal coding.

The question arises as to why place cell-like activity in RSC has not been reported in previous electrophysiological studies^23, 25^. This may have resulted from the difficulty of measuring the activity from a sufficiently-large sample of superficial RSC neurons in behaving animals using microelectrodes, and the fact that RSC ‘place cells’ are intermixed with cells with non-spatial characteristics; however, it is also possible that the combination of head-fixation and behavioural restriction in the current recording context may have masked more complex responses of these neurons, which might be less similar to hippocampal place cells in a free-behaviour context. These possibilities remain to be investigated.

## Methods

### Animals and surgery

All animal procedures were performed in compliance with protocols approved by the ethical research committee of KU Leuven and the University of Lethbridge. Eighteen male C57BI/6j mice (~22–30 g, ~2–4 months old at the time of surgery, including 4 Thy1-GCaMP6s-GP4.3 mice) were used for this study (eight mice for hippocampus electrophysiology, three mice for hippocampus imaging, seven mice for RSC imaging). Mice were injected with dexamethasone (3.2 mg kg^−1^, intramuscular) and anesthetized with isoflurane (1–1.5%, O_2_: 0.5–1 l min^−1^) and body temperature was maintained at 37 °C. A titanium head-plate was attached to the skull^31^ using adhesive cement (C&B-metabond, Parkell) and acrylic material (TAB 2000, Kerr). For all imaging experiments, a 3-mm craniotomy was made above the left RSC^59^ and covered with artificial cerebrospinal fluid (ACSF). A small slit in the dura was made using fine forceps, and a solution containing 1 µl AAV2.1/Syn.GCaMP6m^60^ and 0.3 µl sulforhodamine SR101 was injected with a bevelled micropipette at 0.5 mm ML axis and 1 mm from transverse sinus at two depths (250 and 600 µm deep). At each depth, ~200 nl solution was injected at low speed (9.2 nl per pulse, 15 s interval) using a nanolitre injector (Nanoject II, Drummond Sci.). A cranial window made of three round coverslips (affixed with optical adhesive NOA71, Norland) was implanted and attached to the skull with Vetbond (3M)^31^. For hippocampus imaging, a 3-mm craniotomy was made above the left hippocampus. Injections were made 1.5 mm below the cortical surface (2 mm AP, 1.8 mm ML). Then a small region of cortex (~3 mm diameter, 1 mm thick) was carefully aspired with vacuum. Aspiration was stopped when the cortical white matter was exposed. The hippocampal cranial window was composed of a 1.5 mm-long segment of a glass cylinder (3 mm OD, 2.4 mm ID) with a 3 mm coverslip attached to one end (affixed with optical adhesive NOA71, Norland). The window was inserted into the craniotomy until it touched the white matter and was lightly pushed against it to reduce brain motion and sealed with Vetbond and dental acrylic. The top layer was covered with acrylic material mixed with black pigment to block ambient light from the 2-photon microscope. Two rubber rings were attached to the head-plate to form a well to hold distilled water during imaging. After surgery, mice were moved to the home cage and allowed to recover for a minimum of 5 days. Training started 5 days after implantation. For electrophysiological recordings, surgeries were performed after the animals reached asymptotic performance on the treadmill running task. A 1-mm craniotomy was made above the left hippocampus (2 mm AP, 1.8 mm ML) to allow acute insertion of the recording electrode. The craniotomy was covered with sterile ACSF and a coverslip and sealed with Kwik-Cast sealant (WPI). Electrophysiological recordings were performed over the following 1-3 days.

### Treadmill and training

Mice were trained to be head-fixed and to move on a linear treadmill apparatus^28^ consisting of a 150-cm belt made from Velcro material (Country Brook) covered with texture patches (hot glue stripes, foam, and velcro loop). Two three-dimensional printed wheels of 5-cm radius made from polyamide were attached to bearings with a steel shaft to guide the movement of the belt. An aluminium platform was used to support the belt and the animal. The platform was covered with teflon tape (CS Hyde) to minimize friction. An optical encoder (Avago Tech.) with a resolution of 100 pulses per revolution was attached to the shaft of the wheel to monitor belt movement with a precision of 3.14 mm per pulse. For reward delivery, a photoelectric sensor (Omron) mounted under the platform and reflective tape was attached to the belt triggered the opening of an electromagnetic pinch valve and the release of 10% sugar water reward (~2.5 µl). A custom-assembled PCB board equipped with a microcontroller (AT89LP52, Atmel) was used to monitor the photoelectric signal and control the opening and the closing of the valve. Other mechanical parts were obtained from Thorlabs. The encoder and photoelectric signal were acquired via a USB data acquisition board (Measurement Computing). The signals were recorded with Presentation software (Neurobehavioral Systems) and sampled at 10 kHz.

Mice were scheduled to 1 ml water per day and their weights monitored to be maintained to at least 85% of their free-feeding weights. The mice were introduced to the treadmill and head fixation duration was gradually increased from a few minutes to 1 hour per day over a period of two weeks. Reward was delivered after each lap; however, during training, additional reward was given occasionally to encourage running.

### 2-photon imaging

A custom-built 2-photon microscope (Neurolabware) was used. A Ti:Sapphire excitation laser (MaiTai DeepSee, Spectra-Physics) was operated at 920 nm (~20–60 mW laser power at the sample). Laser scanning was controlled by galvo and resonant scanners (Cambridge 6215H and CRS 8K) through a 16× lens (NA = 0.8, Nikon). Green fluorescence from GCaMP6m was collected using a band-pass filter (510/84 nm, Semrock) with a GaAsP photomultiplier tube (PMT, Hamamatsu). Images were collected at ~30 fps. Imaging sessions normally lasted 10–16 min. Imaging depths were targeted at 150 µm for superficial agranular RSC neurons, and at 400 µm for deep and superficial granular RSC neurons. Layer IV, which was not labelled, was used as reference to distinguish superficial and deep granular RSC neurons. Blackout fabric (Thorlabs) was used to prevent stray light from entering the objective and PMTs.

### Electrophysiology

On the day after the craniotomy, mice were head-fixed and the Kwik-Cast seal﻿ant and coverslip were removed. The craniotomy was rinsed 3-times with ACSF and a small slit was made in the dura. A 4-shank 32-channel acute silicon probe (NeuroNexus) was attached to a micromanipulator (Scientifica). The shanks were at 45° angle (relative to anterior-posterior axis) and lowered into dorsal CA1 (1.2–1.5 mm below pia) at 1 µm per step. The craniotomy was then covered with 2.5% agar (in ACSF) at body temperature. A 256-channel DigiLynx system (Neuralynx) was used to record electrophysiological signals (sampling rate at 32 kHz). Recording started ~20–30 min after insertion. After recording, the probe was retracted slowly and the craniotomy was rinsed with ACSF and covered with a coverslip followed by Kwik-Cast sealant. Recording were performed no more than 3 times in each mouse.

### Data analysis

Data were analysed with MATLAB (The Mathworks, Natick, MA). Images were corrected for motion using TurboReg^61^. Regions of interest (ROI) were identified using morphometric filters^62^. Neurons that were active during the acquisition session were identified by local correlation of 3 × pixels through time. Correlation threshold was set at 0.95 to select active neurons. The baseline-subtracted d*F*/*F*0 was calculated for each ROI using the average across pixels corresponding to that ROI^63^. The time courses were deconvolved to infer underlying firing rates^64^. A method based on constrained nonnegative matrix factorization was used to model calcium transients, from which the count of transients was calculated^32^. For electrophysiological data, raw signals were high-pass filtered (0.8–5 kHz) for spike detection. Spike sorting was done semi-automatically for each probe shank using KlustaKwik followed by manual adjustment^65^. Clusters with clear refractory periods and stable features through time were included for further analysis.

### Place field analysis

Place fields in imaging experiments were identified based on the deconvolved time courses. The 150-cm track was divided into 100 position intervals (bins) The activity was then averaged over the position bins. The results were normalized by the occupancy (i.e., divided by the number of samples) in each position bin. Neurons with mean position activity <0.03% d*F*/*F* s^−1^ were excluded from following analysis. Data were filtered with a Gaussian smoothing window with 4.5 cm s.d. width and lap-to-lap position activity maps were generated. Position activity maps were averaged across trials to obtain position tuning curves. We implemented two approaches to identify place cells: one was based on place field definition and the other used spatial information (SI). First, criteria for place cell selection were adapted from previous literature^30, 33^. Briefly, each place cell had to satisfy the following criteria: (1) Initial threshold was set at 30% of the difference between highest and lowest activity of the position tuning curve. Place fields must be a continuous region with minimum 15 cm width and maximum 120 cm width. (2) The mean in-field activity must be at least three times larger than the mean out-of-field activity. (3) The peaks of the position activity map across trials must be within the potential field for at least one-third of all trials. Neurons that met these criteria were selected as potential place cells. We also used SI criteria to select place cells^66^. SI was calculated as follows:

$${\rm{SI}} = \mathop {\sum}\limits_{i{\rm{ = 1}}}^N {{p_i}\frac{{{f_i}}}{f}{\rm{lo}}{{\rm{g}}_2}\frac{{{f_i}}}{f}} $$

where *p*_*i*_ is the occupancy probability in the *i-*th bin; *f*_*i*_ is the activity in the *i*-th bin (summed activity in that bin divided by the occupancy in that bin); *f* is the overall activity (summed *f*_*i*_ across all bins); and *N* is the total number of bins. Then the time courses were shifted circularly by a random time interval. This process was repeated 1000 times and the distribution of the shuffled SI was constructed. If the original SI was higher than 95 percentile of the shuffled SI, the neuron was considered as significantly spatially tuned. Place-field width was calculated from the number of consecutive position bins in which the mean activity exceeded 20% of the difference between peak and baseline activity. Field widths below 15 cm or above 120 cm were discarded. To confirm that combining datasets from multiple sessions of each animal does not influence the results, we redid the analysis selecting only one dataset from each animal and repeated the same quantification as shown in Fig. 4. The results were comparable to that by combining data sets.

### Population vector analysis

Activity in the position maps was normalized so that the activity of each cell ranged between 0 and 1. For each session, a population vector was constructed using the occupancy-normalized trial-averaged activity of all place cells at each position bin. The population vector correlation matrix was calculated using the pairwise Pearson’s linear correlation coefficient between each pair of columns (population vector at each position bin) in two population vector matrices^35^.

### Data availability

All data reported in this study are available from the corresponding authors upon request.

## Electronic supplementary material

Supplementary Information
