# Supplementary material for: Sparse orthogonal population representation of spatial context in the retrosplenial cortex
Source: Nat Commun. 2017 Aug 15;8:243. doi: 10.1038/s41467-017-00180-9 (PMC5557927; doi:10.1038/s41467-017-00180-9)
Supplement: Supplementary file 1 — Supplementary Information [file 41467_2017_180_MOESM1_ESM.pdf]

File Name: Supplementary Information
Descriptions: Supplementary Figures

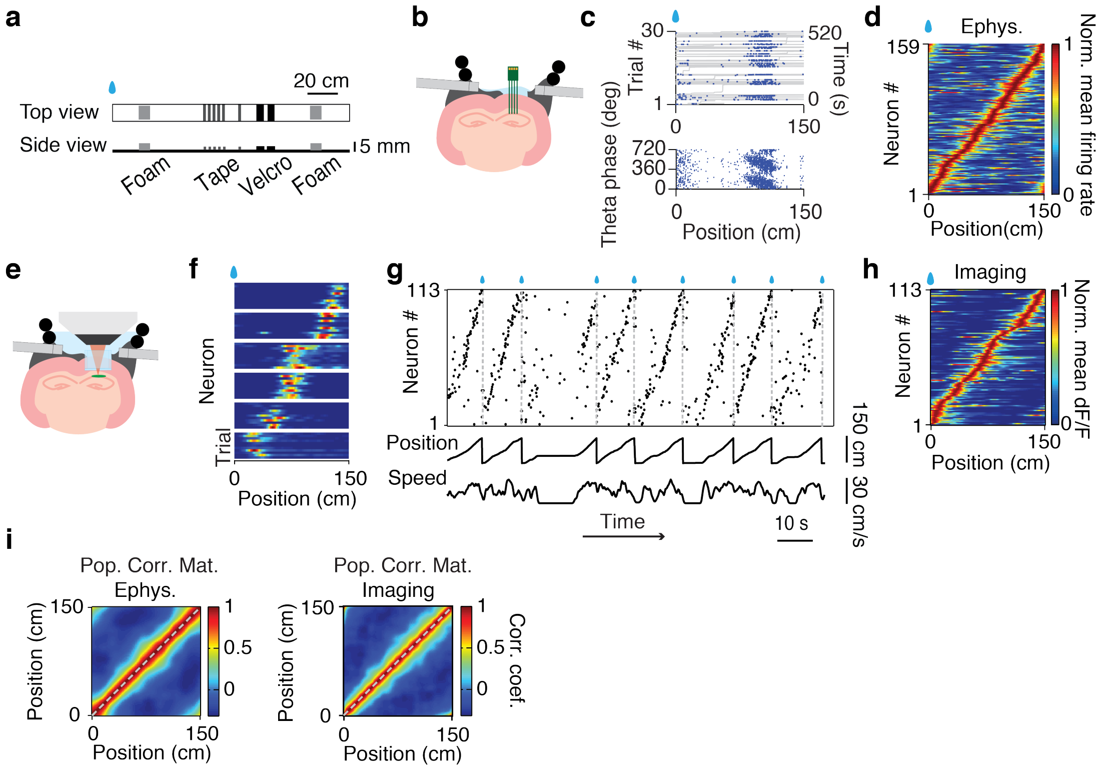

Supplementary Fig. 1. Hippocampal place cells during treadmill locomotion

(a) Detailed belt diagram. The treadmill belt was endowed with tactile cues at distinct locations. Blue drop indicates water reward.

(b) Diagram of multi-shank probe recordings in dorsal hippocampus CA1.

(c-d) Electrophysiology results.

(c) Top: raster of an example CA1 place cell in time vs. track position for 30 trials. Blue dots represent spikes. Gray trace indicates locomotion in position and time. Bottom: scatter plot of the example place cell in theta phase and position. Two theta cycles are shown for illustration purposes.

(d) Trial-averaged normalized firing rates for 159 electrically recorded CA1 place cells from 8 mice ordered by the positions of their peak firing.

(e) Diagram of imaging preparation in dorsal hippocampus CA1 with a glass cannula (a coverslip at the bottom of the cannula) inserted.

(f-h) Imaging results.

(f) Normalized activity as function of location for 6 example CA1 place cells from one example imaging session, shown for multiple trials.

(g) A section of the raster plot showing activation time points for 113 simultaneously imaged CA1 place cells, for the same session as in (f), together with position and movement speed (bottom). Activation time points were defined as time points of peak responses in each lap for each neuron. Cells were ordered by the location that evoked largest responses. Note the repeated activation sequences during movement and lack thereof during stillness.

(h) Trial-averaged normalized activity as a function of location for the 113 place cells shown in (g).

(i) Correlation matrices (Pearson correlation coefficient) of population vectors as a function of position for electrophysiological data (left) and imaging data (right).

(Imaging data from WT mice with AAV1-hSyn-GCaMP6m injections)

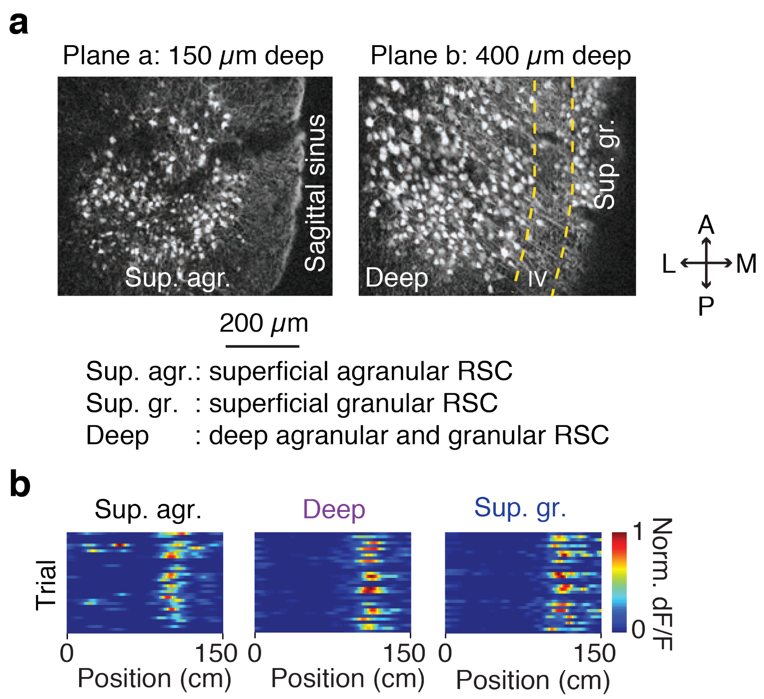

Supplementary Fig. 2. Place cell activity across RSC subregions

(a) Imaging in dorsal (plane a) RSC yielded superficial agranular (sup. agr.) neurons. White blobs highlight active neurons during the run. Imaging in ventral RSC yielded deep neurons and superficial granular (sup. gr.) neurons. Layer IV (between yellow dashed lines) was not labeled. The midline superior sagittal sinus was visible in the field of view.

(b) Position activity maps of 3 example RSC place cells from each of the 3 RSC subregions. (Data from WT mice with AAV1-hSyn-GCaMP6m injections)

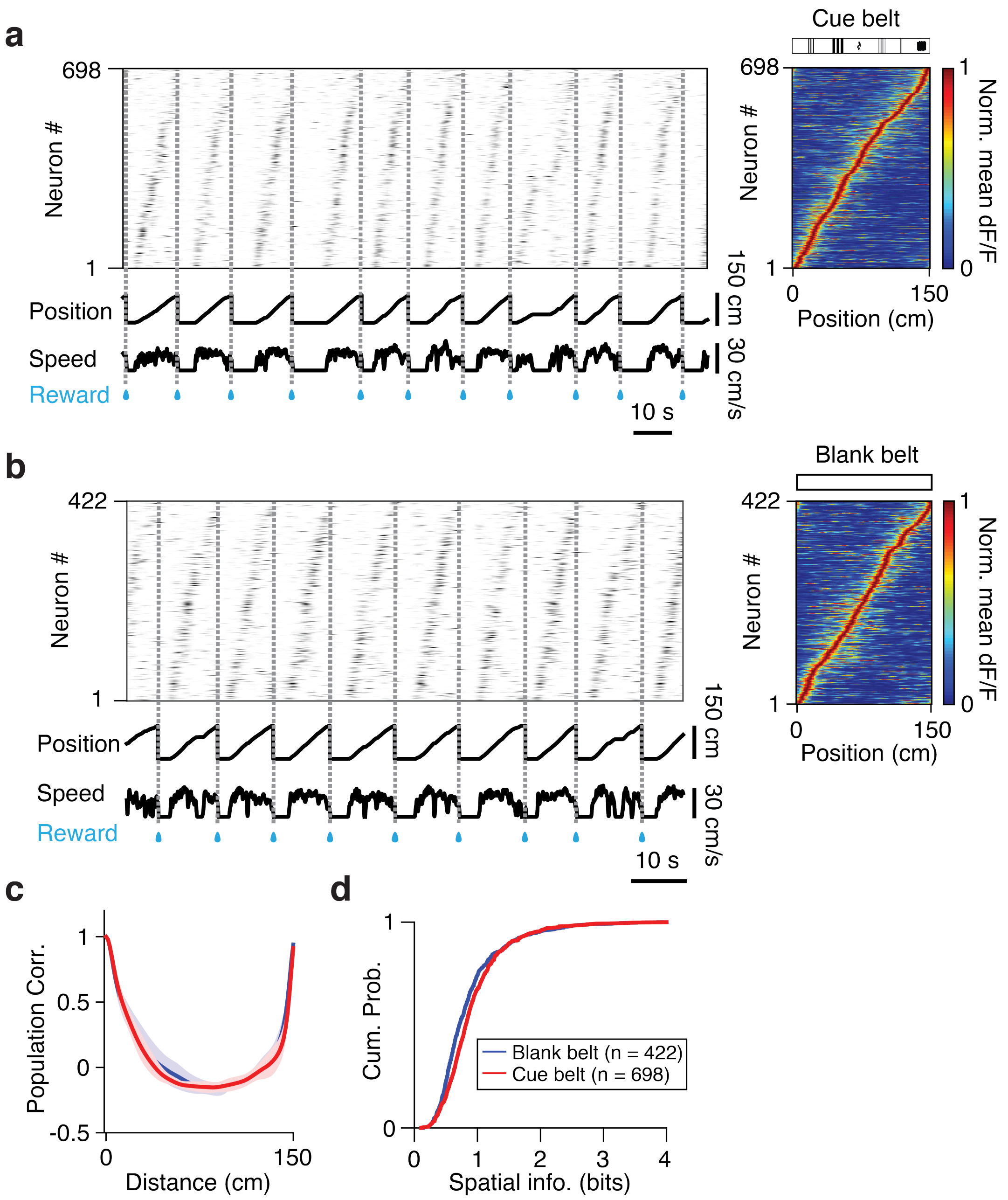

Supplementary Fig. 3. Hippocampal place cell activity on the belts with and without tactile stimuli

(a) Left, a section of the normalized fluorescence activity of 698 simultaneously imaged CA1 place cells on a belt with tactile cues. Cells were ordered by the positions of their peak average activity. Position and speed traces are shown below. Dashed lines and blue drops represent reward delivery. Right, trial-averaged position activity for the 698 RSC place cells shown on the left. Belt diagram is shown at the top.

(b) Left, a section of the normalized fluorescence activity of 422 simultaneously imaged RSC place cells on a belt without any tactile cues. Cells were ordered by the positions of their peak average activity. Position and speed traces are shown below. Dashed lines and blue drops represent reward delivery. Right, trial-averaged position activity for the 135 RSC place cells shown on the left. Belt diagram is shown at the top.

(c) Population vector correlation (Pearson correlation coefficient) as a function of distance for all CA1 place cells on the cue belt (red) and on the blank belt (blue). Shaded areas represent SD.

(d) Cumulative distribution of spatial information for all CA1 place cells on the cue belt (red) and on the blank belt (blue).

(Data from Thy1 GP4.3 transgenic mouse)

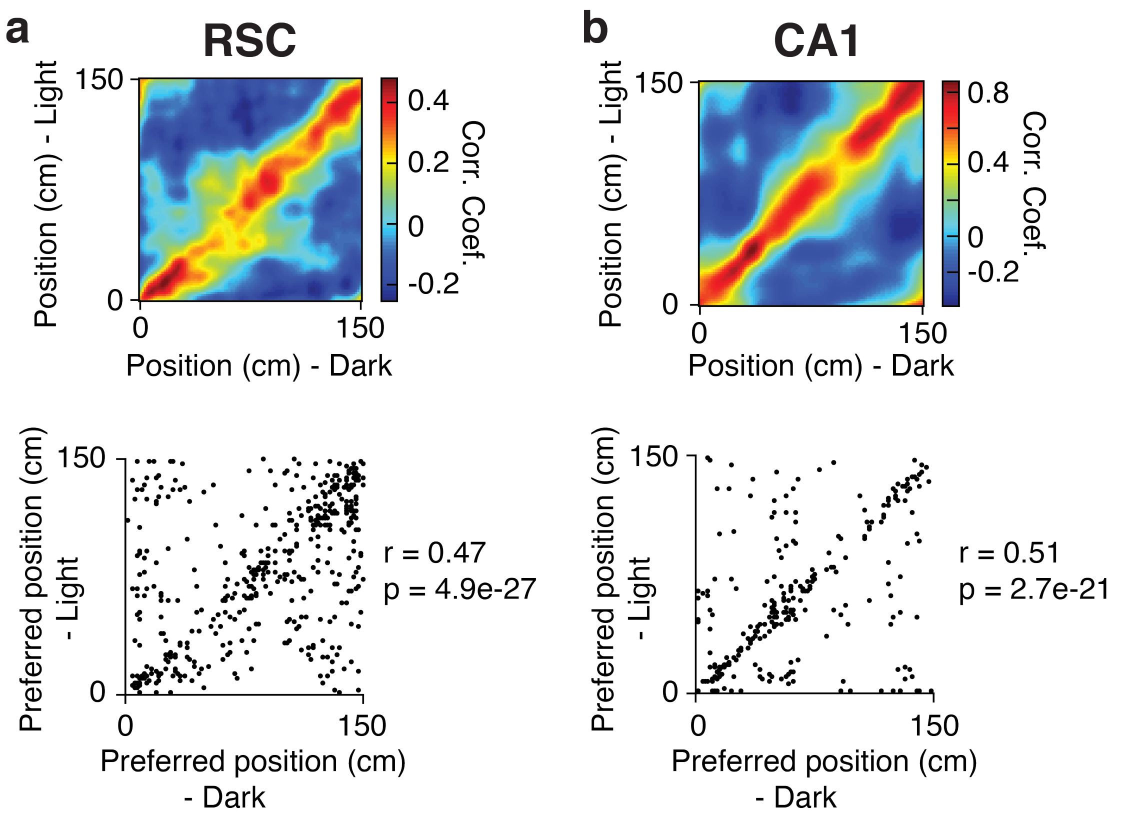

Supplementary Fig. 4. Correlated population representations of position upon illumination change for both RSC and CA1 place cells

(a) Top, population vector correlation (Pearson correlation coefficient) matrix for all RSC place cells under the light and dark conditions. Bottom, scatter plot of the preferred positions for all RSC place cells under the light and dark conditions (n = 458). The correlation coefficient and p value are indicated on the right. Data from Thy1 transgenic mice.

(b) Top, population vector correlation (Pearson correlation coefficient) matrix for all CA1 place cells under the light and dark conditions. Bottom, scatter plot of the preferred positions for all CA1 place cells under the light and dark conditions (n = 288). The correlation coefficient and p value are indicated on the right. Data from WT mouse with AAV1-hSyn-GCaMP6m injections.

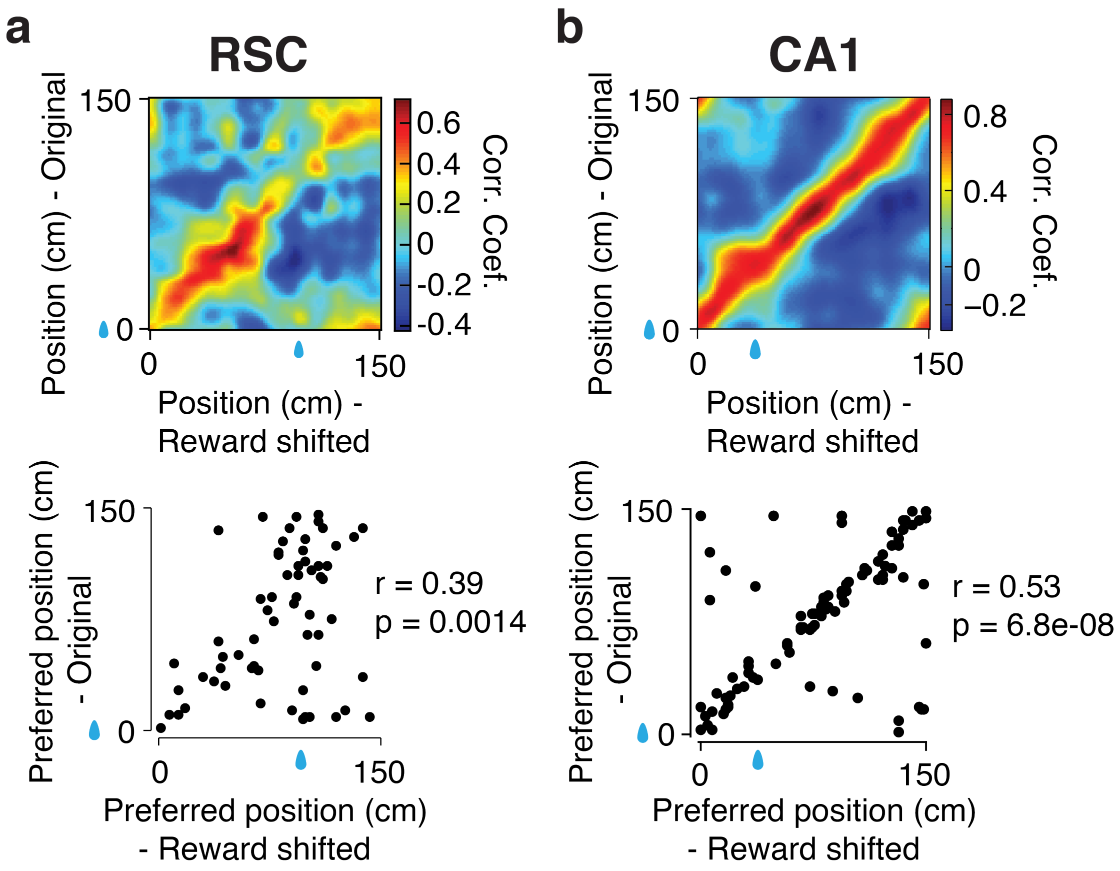

Supplementary Fig. 5. Correlated population representations of position upon reward relocation for both RSC and CA1 place cells

(a) Top, population vector correlation (Pearson correlation coefficient) matrix for all RSC place cells under the conditions of original and shifted reward locations. Blue drops indicate reward locations. Bottom, scatter plot of the preferred positions for all RSC place cells upon reward relocation (n = 63). The correlation coefficient and p value are indicated on the right. Blue drops indicate reward locations. Data from WT mice with AAV1-hSyn-GCaMP6m injections.
(b) Top, population vector correlation (Pearson correlation coefficient) matrix for all CA1 place cells under the conditions of original and shifted reward locations. Blue drops indicate reward locations. Bottom, scatter plot of the preferred positions for all CA1 place cells upon reward relocation (n = 91). The correlation coefficient and p value are indicated on the right. Blue drops indicate reward locations. Data from electrophysiology recordings.
